# Epidermal growth factor (EGF) receptor-ligand based molecular staging predicts prognosis in head and neck squamous cell carcinoma partly due to deregulated EGF- induced amphiregulin expression

**DOI:** 10.1186/s13046-016-0422-z

**Published:** 2016-09-26

**Authors:** Jian Gao, Camilla H. Ulekleiv, Trond S. Halstensen

**Affiliations:** Department of Oral Biology, University of Oslo, P.b. 1052, Blindern, Oslo, 0316 Norway

**Keywords:** Oral squamous cell carcinoma (OSCC), Amphiregulin (AREG), Epidermal growth factor (EGF), Cisplatin resistance

## Abstract

**Background:**

Increased expression of epidermal growth factor receptor (EGFR) and its ligands is associated with poor prognosis and chemoresistance in many carcinoma types, but its role in head and neck squamous cell carcinoma (HNSCC) is unclear. Our aim was to clarify whether mRNA expression of EGFR-ligands was linked to prognosis and cisplatin resistance, and if so, which ligand was most important and how was the expression regulated.

**Methods:**

To examine the prognostic effect of EGFR-ligand expression, we analyzed tumorous mRNA expression in 399 HNSCC patients. The intracellular signaling pathways controlling epidermal growth factor (EGF)-induced amphiregulin (AREG) expression were examined in three oral squamous cell carcinoma (OSCC) cell lines. Effect of AREG on cisplatin resistance was examined by viability assays in four-, and by association in 11 OSCC cell lines.

**Results:**

The patients were divided into five groups according to the median mRNA expression levels of four EGFR ligands, i.e. AREG, EGF, heparin-binding EGF-like growth factor (HBEGF) and beta-cellulin (BTC). The number of increased-expressed EGFR-ligands were progressively correlated to five-year survival, even in advanced TNM-stage IV patients, where five-year mortality increased from 26 % if tumor expressed none to one EGFR-ligand, to 45 % in three to four ligand expressing tumors. Thus, staging the tumor according to these EGFR-ligand mRNA expression pattern completely out performed TNM staging in predicting prognosis. Multivariate analysis identified AREG as the dominating predictor, and AREG was overexpressed in OSCC compared to tumors from other sites. Both EGF and HBEGF stimulation induced strong AREG increase in OSCC cell lines, which was partially mediated by the extracellular signal-regulated kinase 1/2 pathway, and negatively regulated by p38, c-Jun N-terminal kinase, and phosphoinositide-3 kinase. Although increased AREG mRNA expression predicted unfavorable prognosis in platinum treated HNSCC patients, AREG did not mediate cisplatin resistance in the OSCC cell lines.

**Conclusions:**

Increased tumorous mRNA expression of four EGFR ligands was progressively associated with poor prognosis in HNSCC. Thus, EGFR-ligands mRNA expression pattern may be a new prognostic biomarker. The tightly regulated EGF-induced AREG mRNA expression was partly lost in the OSCC cell lines and restoring its regulation may be a new target in cancer treatment.

**Trial registration:**

Not applicable as the clinical data of the 498 HNSCC patients and their mRNA expression profiles were collected from the open TCGA database: http://cancergenome.nih.gov/cancersselected/headandneck.

## Background

Head and neck squamous cell carcinoma (HNSCC) is the sixth most common carcinoma globally [1], with the oral cavity, pharynx and larynx as the most common sites [2]. Despite advances in new therapies, five-year survival rate still remains ~50 % [1].

The majority of epithelial malignancies, including HNSCC have increased expression and activation of epidermal growth factor receptor (EGFR) [3], which is associated with poor prognosis and resistance to chemo-and radio-therapy [4]. The EGFR ligands: epidermal growth factor (EGF), transforming growth factor-α (TGFA), heparin-binding EGF-like growth factor (HBEGF), amphiregulin (AREG), beta-cellulin (BTC), epiregulin (EREG) and epigen (EPGN), may increase tumor growth, invasion, and metastasis through EGFR activation [5]. These ligands and/or the receptor are often deregulated in cancers [6], resulting in increased tumor survival through auto- or paracrine stimulation [7].

Among the ligands, AREG modulates cell proliferation, apoptosis and migration of different cell types including epithelial cells, fibroblasts and immune cells [8] by binding to and inducing EGFR homo-, or hetero-dimerization with ErbB2, ErbB3 or ErbB4. This EGFR activation triggers an intracellular signal cascade through both the mitogen- activated protein kinases (MAPK) pathways, the extracellular signal-regulated kinase (ERK)1/2, the Jun N-terminal kinase (JNK), the p38, and the phosphoinositide 3-kinase/protein kinase B (PI3K/Akt) pathways [9].

Although AREG was first described to inhibit growth of aggressive carcinoma cell lines [10], it is now defined as an oncogenic factor as it is up-regulated and related to poor prognosis in a wide variety of carcinomas, including ovarian, pancreatic, colorectal, breast, pulmonary, bronchial and bladder carcinomas [11], but its role in HNSCC remains controversial. Whereas AREG acts as a tumor promoter for oral squamous cell carcinoma (OSCC) cells [12], and OSCC tissues have higher AREG mRNA level than normal gingivae [13], HNSCC patients have lower AREG serum levels than healthy controls [14]. Despite conflicting results on cell lines, increased cancerous AREG expression has been associated with radiotherapy resistance in pancreatic cancer cell lines [15] and in K-RAS mutated cancer cells, probably by activating the PI3K-Akt survival pathway [16]. Moreover, increased AREG expression may be linked to cisplatin resistance in mammary cancer [17] and HepG2 hepatoma cell lines [18], but not in pulmonary cancer [17] or HNSCC cell lines [19].

Numerous endogenous and exogenous stimuli (EGF, interleukin 1α, tumor necrosis factor-β, gastrin, insulin etc., for reference, see review [11]) may induce AREG in cancer cells, but the intracellular signaling pathway controlling AREG expression is rather unknown. In our study, EGF and HBEGF induced strong AREG increase, which then was used to investigate the intracellular signaling pathways controlling AREG expression. As MAPK and PI3K play a central role in transduction of signals from EGFR, and the activation of Ras/Raf/MAPK pathway induces AREG transcription in colon cancer cells [20], we examined if EGF induce AREG through these pathways. Moreover, HNSCC mRNA expression of AREG, EGF, HBEGF and BTC was correlated to five-year mortality by analyzing data in the cancer genomic atlas database (TCGA, http://cancergenome.nih.gov/). Finally, we examined whether AREG expression or stimulation influenced cisplatin sensitivity in our OSCC cell lines.

## Methods

### Clinical data and RNA expression analysis

We searched and downloaded the mRNA expression profiles and clinical data of 498 HNSCC patients from the TCGA database: (http://cancergenome.nih.gov/). All patients, diagnosed and treated during 1997–2014, were followed until September 30th, 2014. For detailed tumor sample acquisition, see reference [21]. Briefly, tumor specimens were collected at the time of surgical resection. The patients had received no chemo-or radiotherapy prior for their disease. Cases were staged according to the American Joint Committee on Cancer (AJCC), Seventh Edition [22]. mRNA expression profiles were estimated by normalizing raw counts of mapped RNA-sequences reads to human reference genes, and mRNA levels measured as fragments per kilobase per million mapped reads (FPKM). Patients without follow-up data or who died within two months of operation were excluded, and finally 399 patients were included in the study.

### Reagents

Cisplatin, human recombinant EGF, amphiregulin and phosphatase-conjugated anti-rabbit IgG antibody were obtained from Sigma-Aldrich (St. Louis, Missouri, USA). Anti-human phospho-Erk1/2, phospho-p38, phospho-JNK, phospho-ErbB2 (Tyr1248), Erk1/2, p38, JNK, Akt, ErbB2 and EGFR antibodies were obtained from Cell signaling (Beverly, Massachusetts, USA). Human recombinant HBEGF and human amphiregulin ELISA Duoset were purchased from R&D Systems (Minneapolis, MN, USA). Anti-human phospho-Akt (Ser 473) antibody and EGF Receptor (activated) antibody were obtained from Santa Cruz (Dallas, Texas, USA) and Chemicon (Billerica, MA, USA), respectively. Phosphatase-conjugated anti-mouse IgG antibody was purchased from Dako (Glostrup, Denmark).

EGFR inhibitor AG1478 and ErbB2 inhibitor AG825 were obtained from Sigma-Aldrich; MEK inhibitor PD98059, p38 inhibitor SB203580, JNK inhibitor SP600125, and PI3K inhibitor LY294002 were obtained from Calbiochem (Billerica, MA, USA). All the inhibitors were diluted in DMSO (Sigma-Aldrich). The specificity of kinase inhibitors (except for AG825 and PD98059) has been checked through in vitro radiometric filter binding assays by the National Centre for Protein Kinase Profiling, MRC Protein Phosphorylation Unit, University of Dundee, Scotland, UK (http://www.kinase-screen.mrc.ac.uk). The selective EGFR inhibitor AG1478 may, in addition inhibit HER4, however, this did not influence our results as HER4 is not expressed in the cell lines [23].

### Cell lines

Eleven human HNSCC cell lines were used in the study. PE/CA-PJ15 clone B11 (male, 45 years), PE/CA-PJ46 clone B5 (male, 63 years), and PE/CA-PJ49 clone D12 and clone E10 (male, 55 years) were established from tongue tissue; PE/CA-PJ34 clone C12 (basaloid type of OSCC, male, 60 years) and PE/CA-PJ41 clone D2 (female, 68 years) were derived from the oral cavity and the oral squamous epithelium, respectively. The cisplatin resistant subclones, C12cis and D2cis, were selected by exposure to sequential cycles of cisplatin for eight months, which mimic the way the drug is used in the clinic [24]. The above six original cell lines (a kind gift from Dr. A. Berndt and Dr. H. Kosmehl, Friedrich–Schiller University, Germany) and two *in house* made, cisplatin resistant cell lines were cultured under standard condition as previously described [24]. The remaining three cell lines, H376 (female, 40 years) from floor of the mouth, H413 (female, 53 years) from the buccal mucosa and SCC9 (male, 25 years) from tongue (all from ECACC, Salisbury, UK), were cultured in Dulbecco’s modified Eagle’s medium: Ham’s F12 (1:1) (Sigma-Aldrich), 2 mM L-Glutamine, 10 % fetal bovine serum (FBS), 0.5 μg/ml sodium hydrocortisone succinate (Sigma-Aldrich) and penicillin-streptomycin, at 37 °C and 5 % CO_2_.

### Cell viability assay

Cells were seeded at a density of 4000 cells per well in 96-well microtiter plates (Nunc, Wiesbaden-Biebrich, Germany) in 100 μl culture medium with 10 % FBS per well in quintuplicate. After 24 h, culture medium was exchanged to medium with 10 % FBS and different concentration of cisplatin or growth factors. Cells were further grown for 72 h, before incubated in 50 μl XTT labeling mixture (Roche Molecular Biochemicals, Mannheim, Germany) for four h, and then scanned at 450 nm in an Epoch Microplate Spectrophotometer (BioTek, Winooski, USA).

### Quantitative reverse transcriptase polymerase chain reaction (qRT-PCR)

Cells were serum-starved overnight and inhibitors or solvent alone were applied one h prior to EGF-stimulation. Cells were stimulated with 25 ng/ml EGF or left unstimulated, for four h prior to harvesting.

Total RNA was extracted using RNeasy kit (QIAGEN, USA), and complementary DNA (cDNA) was synthesized by RT-RTCK-05 kit (Eurogentec, Berlin, Germany) and stored at −20 °C. A standard real-time PCR reaction with SYBR green Real MasterMix (Eppendorf, Hamburg, Germany) was performed in duplicates using Mx3005p (Agilent Technologies, USA) under the following conditions: 95 °C for 2 min followed by 40 cycles of 95 °C for 20 s, 60 °C for 1 min and 68 °C for 30 s. Dissociation curves ensured product uniformity. Expression data was normalized to the housekeeping gene TATA-box binding protein (TBP). The relative expression levels of the gene of interest were calculated using the 2^-ΔΔCt^ method. AREG primers were obtained from Sigma-Aldrich: forward 5′-GCT-CAG-GCC-ATT-ATG-CTG-CTG-3′, reverse 5′-ACT-CAC-AGG-GGA-AAT-CTC-ACT-CC-3′; TBP primers were obtained from Eurogentec: forward 5′-CGT-GGC-TCT-CTT-ATC-CTC-ATG-A-3’, reverse 5’-GCC-CGA-AAC-GCC-GAA-TAT-A-3’.

### Western blotting

Cells were incubated with low serum medium (0.1 %) for 24 h and inhibitors or solvent alone were applied one h prior to EGF stimulation. Cells were stimulated with 25 ng/ml EGF or left unstimulated for 5 min. then harvested and lysed in CelLytic M Cell Lysis Reagent (Sigma-Aldrich) with protease and phosphatase inhibitor cocktails (Pierce Biotechnology, IL, Rockford, USA). Protein concentrations were determined by the Bio-Rad protein assay (Bio-Rad, Munich, Germany), and 50 μg proteins were separated by 10 % casted sodium dodecyl sulfate–polyacrylamide gel electrophoresis (SDS-PAGE) and electroblotted onto PVDF membranes (Bio-Rad). After BSA (5 %) blocking for one h, the membranes were incubated with primary antibodies overnight at 4 °C. The blots were then washed three times and incubated with secondary antibodies at room temperature for one h, washed three times and visualized with ECF substrate (GE Healthcare, Uppsala, Sweden) in a scanner (Storm, GE Healthcare).

### Enzyme linked immunoassay (ELISA)

The AREG secretion was evaluated using the human amphiregulin ELISA DuoSet (R&D). Cells were seeded in 96-well plates at densities of 6000 cells per well. Duplicate samples were plated for each treatment. The cells were allowed to attach overnight, then medium was changed to culture medium with 0.1 % FBS. After further incubation for 24 h, inhibitors were added one h prior to stimulation, then supernatant was harvested after 48 h and directly used in ELISA assay. Absorbance was read with Epoch Microplate Spectrophotometer (BioTek), and results were analyzed by Gene 5 software (BioTek).

### Statistics

Statistical analysis was performed using Graphpad prism 6.0 (San Diego, California, USA). The survival distributions were compared with the log-rank test (Kaplan–Meier method). Deaths from any cause were defined as events. The patients were censored at loss to follow-up, defined as the last date of contact or at five years after diagnosis. For group differences of normally distributed data, means were compared using Student’s *t*-test for two categories. Where data were not normally distributed, medians were compared using Wilcoxon rank-sum test for two categories or Kruskal-Willis test for more than three categories. Multivariate analysis was performed using Cox Regression method. *p* values < 0.05 were considered significant.

## Results

### EGFR ligands mRNA expression predicted poor prognosis in HNSCC patients

**Fig. 1:**
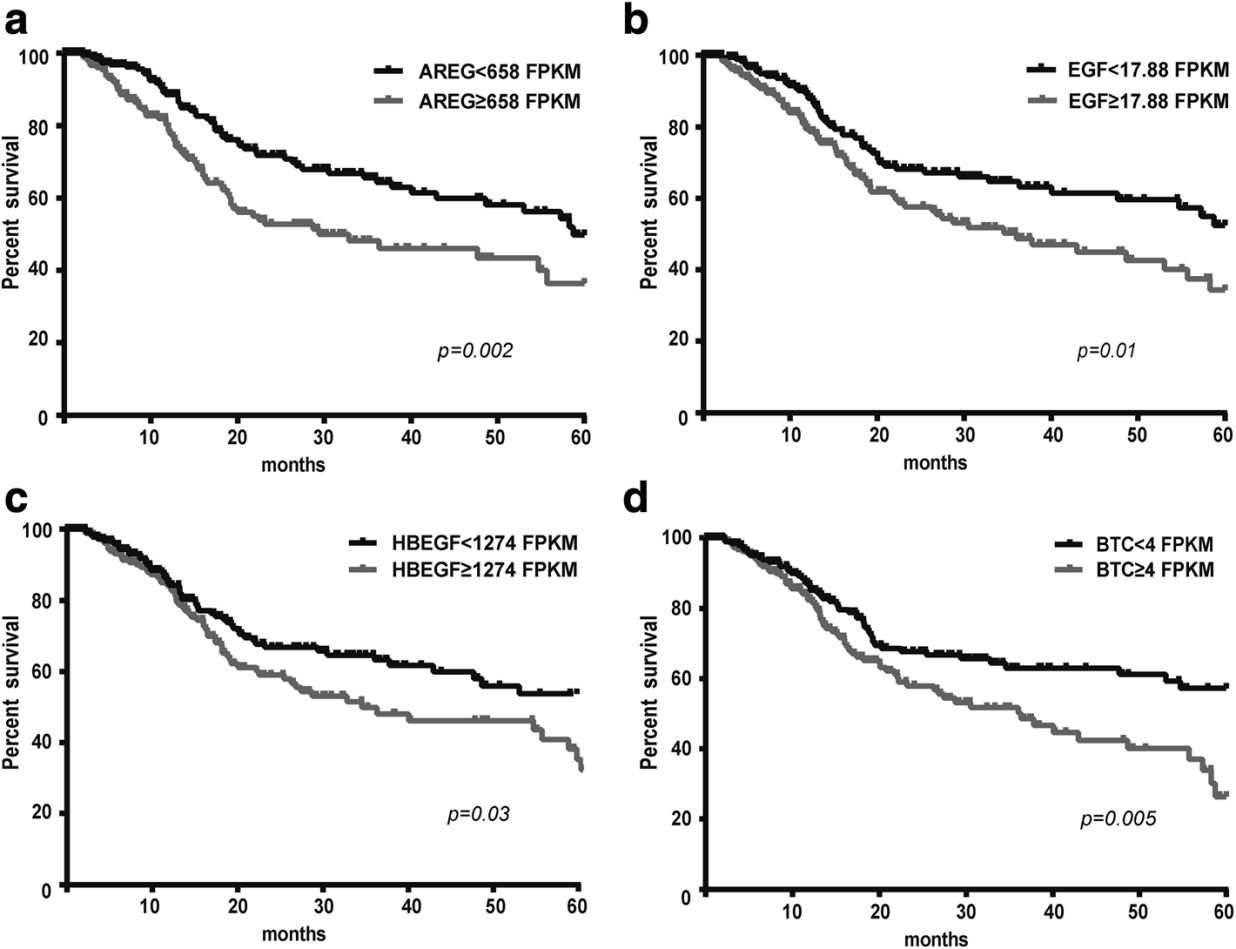
High tumorous mRNA levels of EGFR-ligands predicted poor prognosis in HNSCC patients. HNSCC patients with higher than median tumorous AREG (**a**), EGF (**b**), HBEGF (**c**) or BTC (**d**) mRNA levels had lower five-year survival rate compared to patients with lower than median expression levels (Kaplan-Meier curve, Log rank test). The mRNA levels are measured as fragments per kilobase per million mapped reads (FPKM)

A total of 399 patients, 284 (71 %) men and 115 (29 %) women, median 61 years (range from 19 to 90), were admitted in the study. The detailed patients’ information is shown in Table 1. Dividing patients using median mRNA expression levels as discriminators revealed that increased mRNA expression for four EGFR ligands (AREG, EGF, HBEGF and BTC) was associated with significantly reduced five-year survival compared to patients with lower expression (Fig. 1). Expression of the three other EGFR-ligands (EREG, EPGN and TGFA) was not linked to prognosis.

**Table 1:** Clinical and histological characteristics HNSCC patients in TCGA database

Characteristic	n (%)	*AREG* mRNA level^a^	*p* value^b^
Gender			
Male	284 (71)	1249	0.48
Female	115 (29)	1199	
Age			
≤ 49	68 (17)	238.1	0.28
50–59	112 (28)	193.7	
60–69	133 (33)	193.1	
≥ 70	86 (22)	188.8	
Tumor sites			
Oral Cavity	246 (62)	1441	<0.0001***
Lip	2 (1)	129	
Oropharynx	55 (14)	552	
Larynx	91 (23)	972	
Hypopharynx	5 (1)	1516	
Tumor pathological stage			
I–II	84 (21)	972	0.08
III–IV	259 (65)	1395	
Missing	56 (14)	730	
Tumor pathological T			
T1-T2	141 (35)	1090	0.03*
T3-T4	207 (52)	1402	
Missing	51 (13)	780	
Tumor pathological N			
N0	130 (33)	1312	0.67
N1-3	183 (46)	1352	
Missing	86 (21)	759	
Tumor histological grade			
G1-2	284 (71)	1296	<0.001***
G3-4	103 (26)	1082	
GX	12 (3)	369	
Smoking history			
Smoker	305 (76)	1216	0.83
Non-smoker	84 (22)	1170	
Unknown	10 (2)	1482	
Alcohol history			
Alcohol consumption	273 (68)	973	0.24
No alcohol consumption	119 (30)	1309	
Unknown	7 (2)	1517	
Human papillomavirus			
Positive	18 (4)	55	<0.001***
Negative	66 (17)	807	
Unknown	315 (79)	1391	

^a^mRNA expression levels were measured as fragments per kilobase per million mapped reads (FPKM), medians were shown

^b^Group differences were compared using Wilcoxon rank-sum test or Kruskal-Willis test (*p < 0.05, ***p < 0.001)

**Fig. 2:**
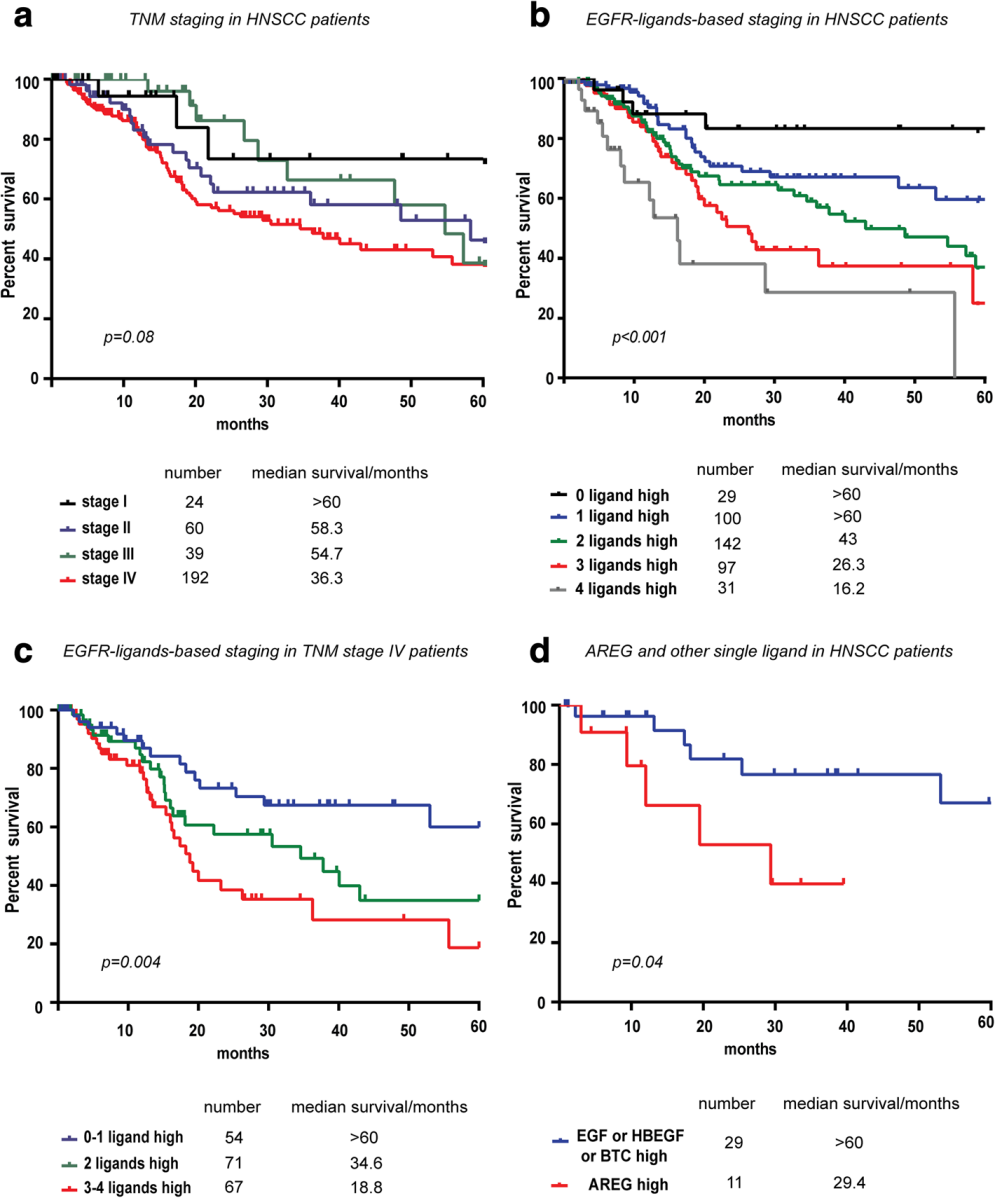
EGFR-ligands-based molecular staging outperforms the TNM system in predicting patient prognosis. **a** The TNM staging was unable to predict patients five-year survival (Kaplan-Meier curve, Log rank test, *p* = 0.08). **b** Expressing more than median mRNA levels of either none, one, two, three or four of the EGFR ligands (i.e. AREG, EGF, HBEGF and BCT) was significantly linked to reduced five-year survival (Kaplan-Meier curve, Log rank test, *p* < 0.001). Whereas only 14 % of the patients (4/29) with tumors that expressed less than median mRNA levels of the four EGFR ligands died within five years, the number of death increased as the number of higher-than-median expressing ligands increased to one (25 %, 25/100), two (31 %, 44/142), three (35 %, 34/97) or four (45 %, 14/31). **c** EGFR-ligand mRNA expression predicted patient survival with TNM stage IV disease (Kaplan-Meier curve, Log rank test, *p* = 0.004). Whereas only 26 % (14/54) of patients died within five years if the OSCCs expressed increased mRNA for none or one of the EGFR-ligands, it increased to 32 % (23/71) and 45 % (30/67) if the tumors expressed increased mRNA for two, or three to four EGFR-ligands, respectively. **d** Patients with over-median-AREG-expressing tumors had worse prognosis than patients expressing any of the other three ligands in the single-ligand-expressing group (Kaplan-Meier curve, Log rank test, *p* = 0.04)

Standard algorithms for TNM staging in HNSCC patients did not predict prognosis, and patients in stage II and III had similar five-year survival (Fig. 2a). Since the mRNA expression levels of these four EGFR-ligands predicted patients outcome separately, we examined if the number of EGFR ligands with above-median mRNA expression level predicted the prognosis even better. Patients were divided into five groups according to number of ligands with increased tumorous mRNA expression levels. This revealed a dose-effect pattern with decreased survival as a function of the number of ligands that were expressed above median level. Thus, such “molecular EGFR-ligand staging” predicted patient outcome much better than the TNM staging system (Fig. 2b). The same result was observed with patients with TNM stage IV disease. Whereas only 26 % (14/54) of stage IV patients died within five years when the HNSCCs expressed increased mRNA for none or one EGFR-ligand, the number increased to 32 % (23/71) and 45 % (30/67) if the tumors expressed increased mRNA for two or more than three EGFR-ligands, respectively (Fig. 2c). Further analysis in patients with only “one-ligand” tumors revealed that patients with over-median AREG-mRNA expression levels had significantly increased mortality compared to patients with any of the other three “single ligand” expressing tumors (Fig. 2d).

Cox multivariate analysis also revealed that AREG played a predominant role among the four ligands (Table 2). This was further supported in detailed analysis of stage IV patients in which AREG expression was associated with significantly reduced five-year survival (Fig. 2d).

**Table 2:** Univariate and multivariate overall survival analysis of epidermal growth factor receptor (EGFR) ligands in 399 HNSCC patients^a^

	Univariate analysis		Multivariate analysis	
Factors^b^	p	HR (95%CI)^c^	p	HR (95%CI)
*EGF*	0.008	1.635 (1.139–2.348)	0.002	1.776 (1.233–2.559)
*HBEGF*	0.034	1.477 (1.031–2.116)	0.32	1.218 (0.828–1.793)
*TGFA*	0.20	1.583 (0.882–1.806)		
*AREG*	0.002	1.759 (1.227–2.522)	0.001	1.857 (1.257–2.743)
*BTC*	0.006	1.662 (1.157–2.386)	0.002	1.778 (1.233–2.564)
*EPGN*	0.23	0.802 (0.562–1.145)		
*EREG*	0.28	1.218 (0.852–1.757)		

^a^Log-rank test was used in univariate analysis and Cox Regression method was used in multivariate analysis

^b^mRNA expression levels were measured as fragments per kilobase per million mapped reads (FPKM) and median mRNA expression levels were used as discriminators (epidermal growth factor (EGF): 17.88 FPKM; heparin-binding EGF-like growth factor (HB-EGF): 1274 FPKM; transforming growth factor-α (TGFA): 1695 FPKM; amphiregulin (AREG): 658 FPKM; beta-cellulin (BTC): 4 FPKM; epigen (EPGN): 32.5 FPKM; epiregulin (EREG): 350 FPKM), and the groups of patients with lower expression levels were set as reference

^c^HR, Hazard ratio. 95 % CI, 95 % confidence interval

**Fig. 3:**
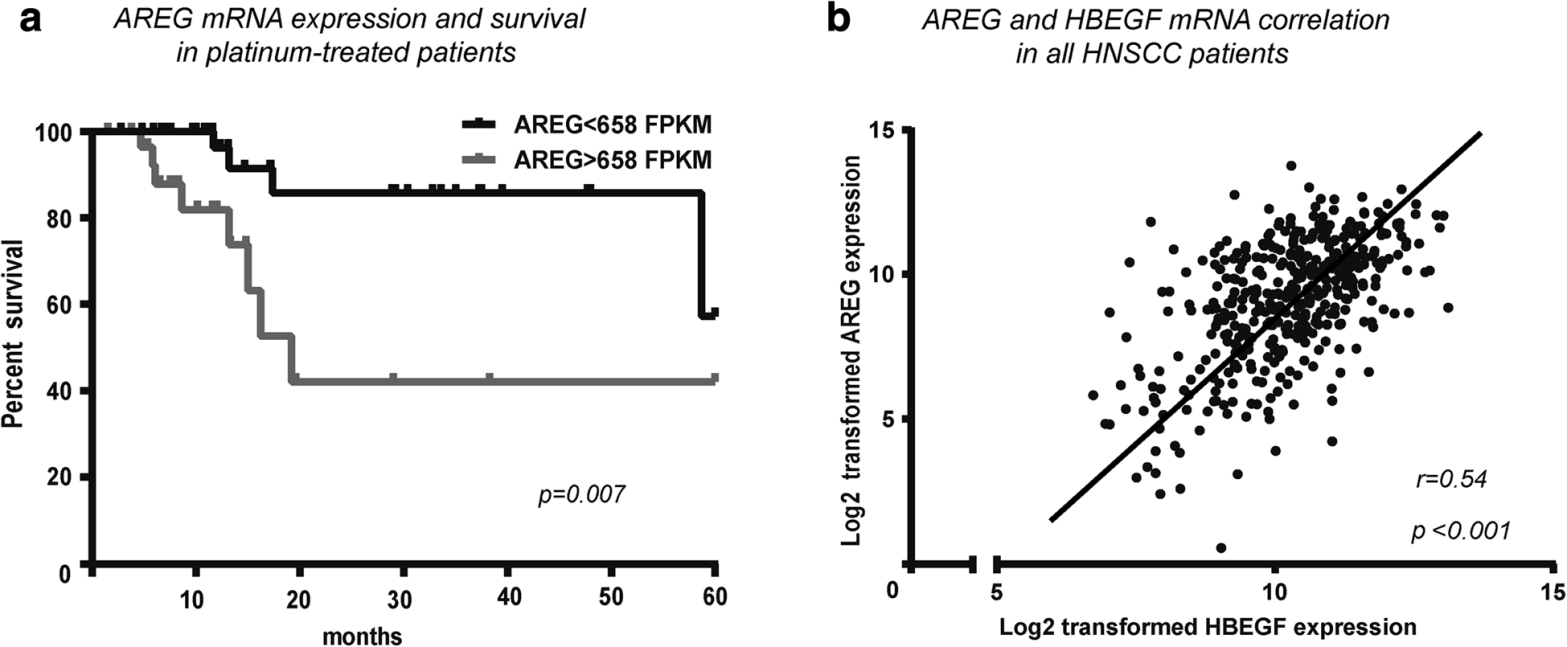
Increased tumorous AREG expressing was associated with poor prognosis and with increased HBGEF expression. **a** Whereas only 7.5 % (3/40) of cis-/carbo-platin treated patients with lower than median tumorous AREG mRNA levels died within 20 months, 26.7 % (8/30) patients with higher levels died (Kaplan-Meier curve, Log rank test, *p* = 0.007). **b** Tumorous AREG mRNA expression was correlated to HBGEF mRNA expression, only (*p* < 0.001; *r* = 0.54, Spearman correlation analysis)

As AREG expression has been associated with cisplatin resistance in many carcinoma types, we examined AREG mRNA expression in the 70 cis-/carbo-platin treated patients and compared it to five-year survival. Patients with high AREG mRNA levels had poor prognosis despite cisplatin treatment (Fig. 3a), while this association was not found with other ligands (not shown). Thus, increased AREG expression may increase cisplatin resistance in HNSCC, as reported for mammary cancer cell lines [17].

### AREG mRNA level was linked to larger, well differentiated and human papillomavirus negative tumors

While risk factors such as smoking and alcohol abuse had no influence on AREG expression level, it was linked to tumor size and differentiation grade as large tumors (T3-T4) had higher AREG expression than smaller tumors (T1-T2) (Table 1), and well-differentiated tumors (G1-G2) had higher AREG expression than poorly differentiated tumors (G3-G4) (Table 1). Human papillomavirus negative (HPV-) tumors also had higher AREG expression than HPV positive (HPV+) tumors (Table 1). Moreover, OSCC had higher AREG expression than SCC from oropharynx and larynx (Table 1), suggesting that AREG expression was particularly deregulated in oral carcinomas. Finally, AREG and HBEGF expression levels were positively correlated in the carcinomas (Fig. 3b).

### EGF-induced Erk1/2, p38, JNK and Akt pathways

**Fig. 4:**
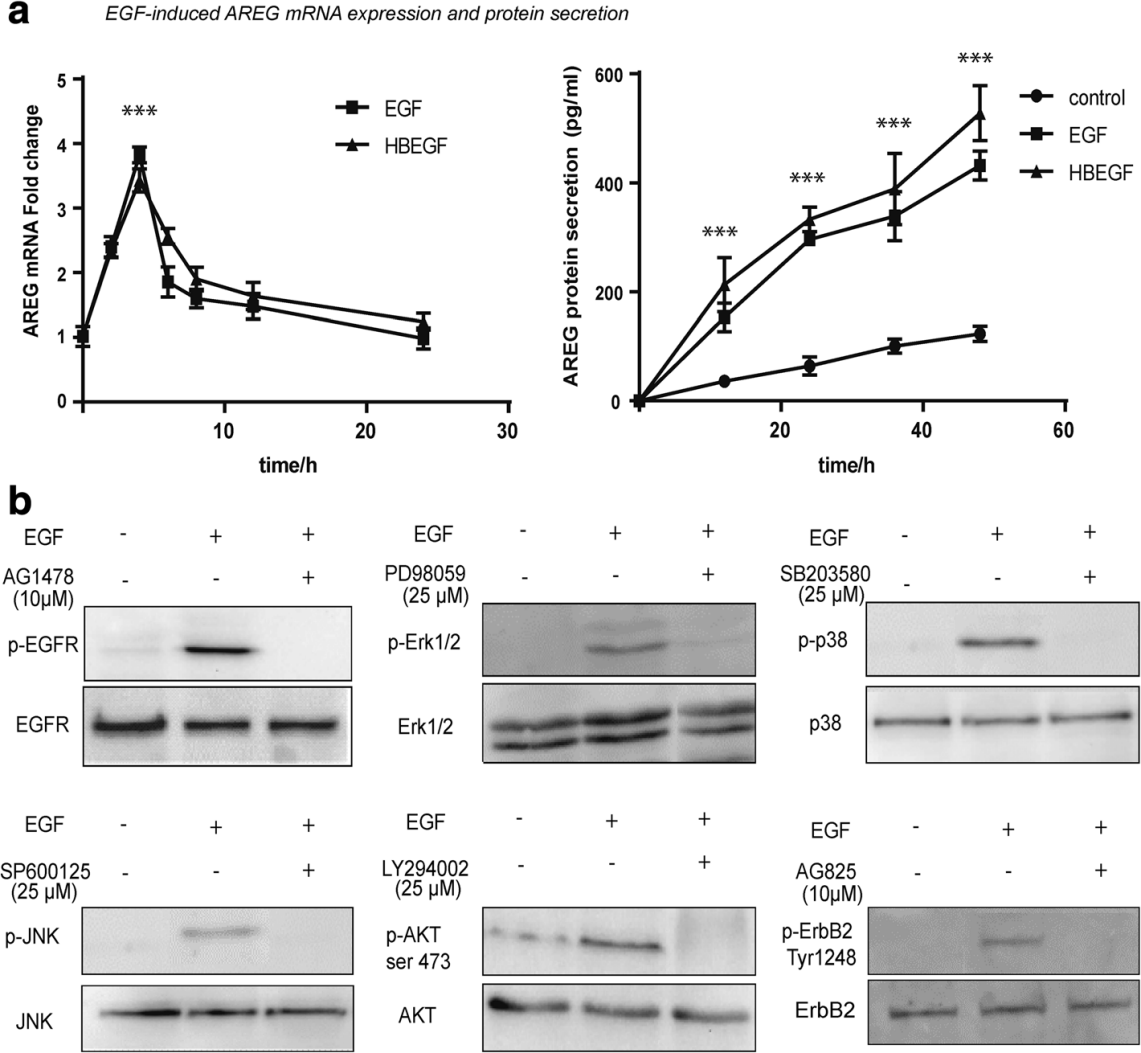
EGF and HBEGF induced AREG expression and actuated MAPK and PI3K/Akt pathways in OSCC cell lines. **a** Both EGF and HBEGF stimulated AREG mRNA expression after two h, peaked at four h and disappeared after 24 h (left panel, Student’s *t*-test, ***p < 0.001). This was accompanied by increased AREG protein concentration in the supernatant during the following 48 h (right panel; untreated cells were used as control group; Student’s *t*-test, ***p < 0.001). A representative experiments in the D2 cell line is shown. **b** EGF induced phosphorylation of the EGF receptor, ERK, p38, JNK, Akt and ErbB2 could be completely inhibited by specific kinase inhibitors. Western blotting from a representative experiment using the D2 cell line

Since cancer extracellular matrix releases EGF and HBEGF [25], and AREG correlated to HBEGF expression, we hypothesized that extracellular EGF and/or HBEGF induced AREG overexpression in OSCC. We therefore treated three OSCC cell lines, C12, D2 and E10, with EGF and HBEGF and observed that both factors induced AREG mRNA and protein increase in the same manner (Fig. 4a). Thus, we used EGF stimulation to examine how AREG overexpression is controlled in OSCC. As shown in Fig. 4b, EGF-stimulation induced Erk1/2, p38, JNK, Akt, EGFR and ErbB2 phosphorylation. The activation of each of these pathways could be blocked by specific kinase inhibitors.

### Inhibiting EGFR signaling pathways affected EGF-induced AREG mRNA expression differently

**Fig. 5:**
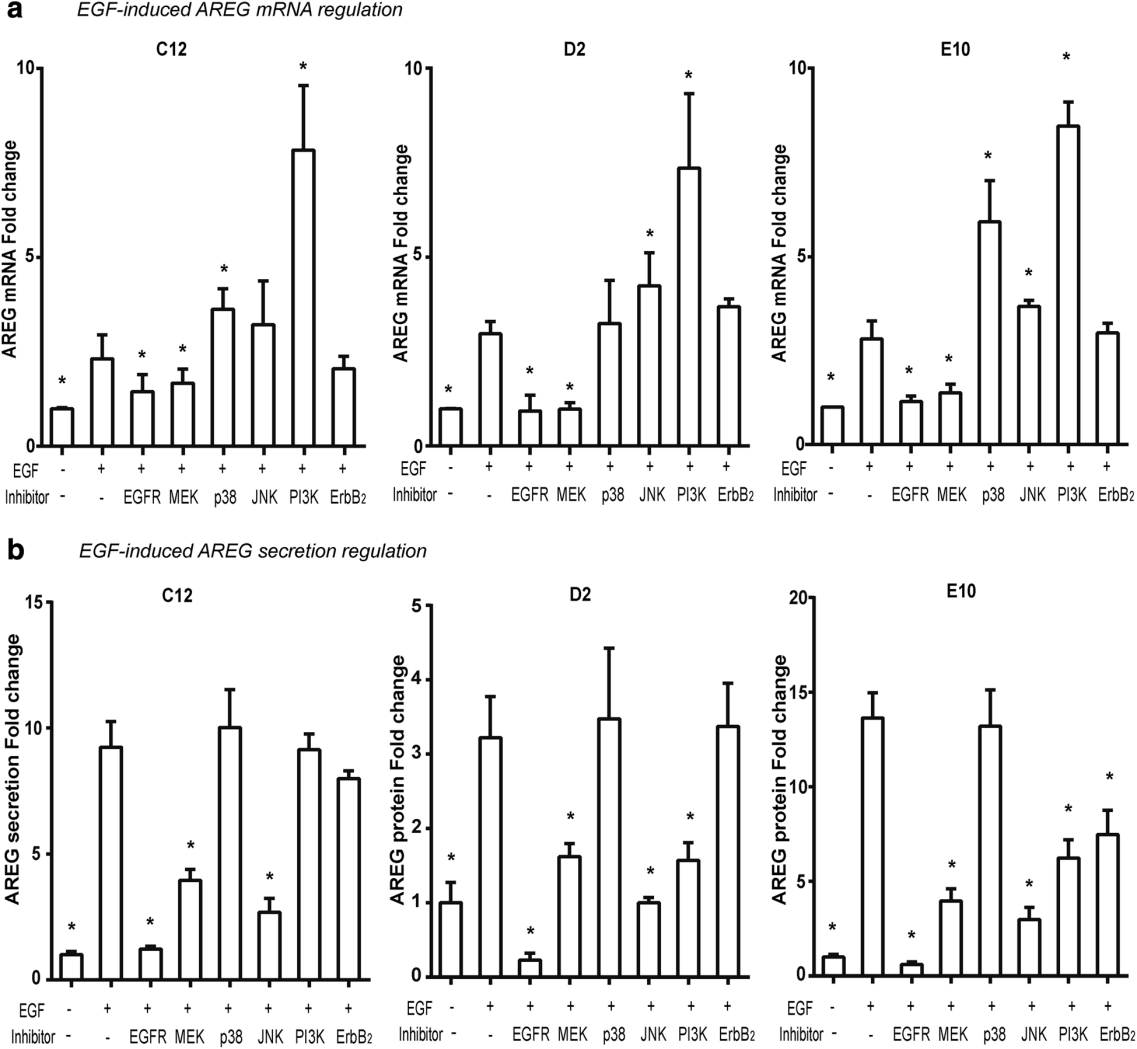
The intracellular pathways in EGF-induced AREG expression. The EGF-induced AREG mRNA (**a**) and protein (**b**) expression was reduced after EGFR-kinase inhibition in all cell lines, while it showed no reduction after ErbB2-kinase inhibition and was differently affected by the MAPK inhibitors. **a** Whereas MEK-inhibition reduced EGF-induced AREG mRNA expression in all the three cell lines, p38- and JNK-inhibition did not. PI3K-inhibition increased EGF-induced AREG expression in all cell lines. **b** EGF-induced AREG protein secretion could be totally blocked by EGFR- inhibition. Whereas MEK- and JNK-inhibition profoundly decrease EGF-induced AREG expression in all cell lines, p38-inhibition had no effect. The PI3K-inhibitor reduced EGF-induced AREG production in the conventional OSCC cell line D2 and E10, but had no effect on the basaloid OSSC cell line C12. The ErbB-2- inhibition inhibited the AREG increase in E10 cell line, only. *represents significant difference with EGF stimulation groups without inhibitors (student’s *t*-test)

AREG mRNA level showed a peak four h after EGF-stimulation (Fig. 4a), which was then used to examine the dynamics in EGF-induced AREG mRNA expression. Whereas the EGFR-kinase inhibitor (AG1478) attenuated EGF-induced AREG mRNA expression, the ErbB2 kinase inhibitor (AG825) did not (Fig. 5a). Whereas the MAPK/ERK kinase (MEK) inhibitor (PD98050) reduced EGF-induced AREG mRNA expression, the PI3K inhibitor (LY294002) increased it in all three cell lines. The p38 inhibitor (SB203580) revealed a differential response pattern as it increased EGF-induced AREG mRNA expression in the cell lines C12 (basaloid SCC) and E10, only. The JNK-inhibition (SP600125) had similar effect but only in the two conventional OSCC cell lines D2 and E10.

### EGF-induced AREG protein secretion was profoundly inhibited by ERK1/2 and JNK pathway inhibitors

The EGFR kinase inhibitor, but not the ErbB2 kinase inhibitor, reduced EGF-induced AREG protein secretion substantially in all cell lines, except the E10 (Fig. 5b). Whereas MEK- and JNK-inhibition reduced AREG secretion in all cell lines, p38 inhibition did not. PI3K-inhibition partly blocked EGF-induced AREG protein secretion in the two conventional OSCC cell lines, but not in the basaloid C12 cell line.

### Exogenous EGF or AREG did not increase cisplatin resistance

**Fig. 6:**
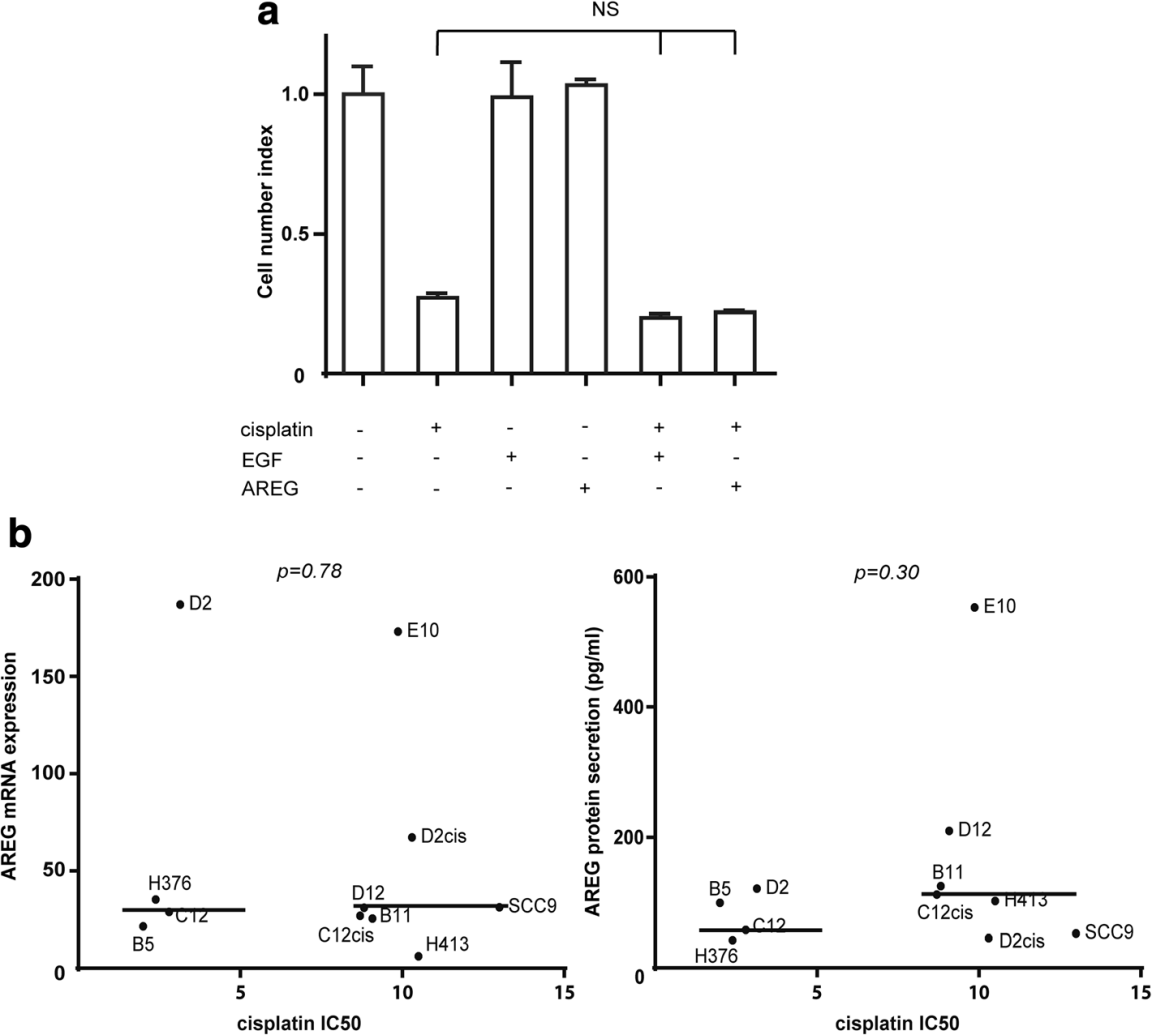
Cisplatin resistance was independent of AREG expression in 11 OSCC cell lines. **a** Neither exogenous EGF nor AREG affected cisplatin resistance in OSCC cell lines. Cells were grown in the presence (+) or absence (−) of human recombinant EGF (50 ng/ml), human recombinant AREG (100 ng/ml) for 24 h and then treated with (+) or without (−) 10 μM cisplatin (NS: not significant. *p* > 0.05, student’s *t*-test). **b** Cisplatin IC50 value did not correlate to AREG mRNA expression (left) or protein production (right) in 11 OSCC cell lines. The cisplatin IC50 value in sensitive (*n* = 4) or resistant (*n* = 7) OSCC cell lines were plotted against AREG mRNA expression (left) or 24 h AREG production (right). The cell lines were clustered into cisplatin sensitive (IC50 < 3.5 μM) or resistant (IC50 > 8.5 μM). Median indicated with horizontal lines (Wilcoxon rank-sum test)

Patients with high AREG expression had poor clinical response to cisplatin treatment (Fig. 3a), suggesting that AREG increased cisplatin resistance in the HNSCC, as revealed for several other carcinoma types [17, 18]. However, neither EGF nor AREG increased cisplatin resistance in any of the four cisplatin sensitive cell lines (Fig. 6a). Moreover, neither AREG mRNA nor protein expression was associated with cisplatin resistance in the 11 OSCC cell lines (Fig. 6b), and cisplatin treatment did not change the AREG mRNA expression or secretion (data not shown).

## Discussion

Pathological based TNM staging uses a combination of primary tumor size (T), regional lymph node spread (N), and distant metastases (M), and is currently the dominating system to determine treatments and prognosis in HNSCC patients. However, the TNM staging system is rather unreliable in predicting prognosis and is, therefore, often combined with biological markers to further subdivide the cancer, such as prostate-specific antigen (PSA) in prostate cancer, estrogen receptor (ER), progesterone receptor (PR), and ErbB2 in breast cancer [26]. The biomarkers reflect fundamental biological cancer features that may predict treatment responses and prognosis better than conventional TNM staging does alone.

Here we report, apparently for the first time, that increased mRNA expression levels of the EGFR ligands (AREG, EGF, HBEGF and BTC) predicted prognosis in an almost dose-ligand number dependent manner, and this predicted prognosis far better than the TNM system.

The EGFR ligands not only have different affinity for the receptor [27], but there is a dose- modulated ligand-specific response [28, 29]. These are partly mediated by ligand-specific phosphorylation of tyrosine residues in the EGFR [30]. The ligands has been placed in three groups based on ensemble clustering of their overall response: Group 1) EGF, AREG and EPR; group 2) BTC, TGFA and EPG; and group 3) HBEGF [29]. This may explain why patients with tumors that expressed increased mRNA levels for EGFR-ligands in all three response groups: Gr.1 (AREG, EGF), Gr.2 (BTC), and Gr. 3 (HBEGF) had particular poor prognosis, as this activated all EGFR-inducible intracellular signaling pathways.

The EGFR was highly expressed in our cell lines [23], despite having only one copy of an un-mutated EGFR gene [31]. Screening of EGFR-ligand mRNA expression revealed that AREG was considerably higher expressed than any other EGF family members (data not shown), similar to what the multivariate analysis revealed in the patients. In addition, patients with increased AREG levels only, had significantly reduced prognosis compared to those with increased EGF, HBEGF or BTC, only. Thus, AREG may be a particular important EGFR ligand in cancer biology.

Cancer associated fibroblasts disrupt extracellular matrix to generate a track for carcinoma cells to follow [32]. Such process releases growth factors (i. e. EGF and HB-EGF), stored in the extracellular matrix, which then would induce increased cancer cell proliferation [25] and EGF/HB-EGF induced AREG expression as shown in this study, which would enhance AREG induced infiltrative behavior and facilitate metastasis.

**Fig. 7:**
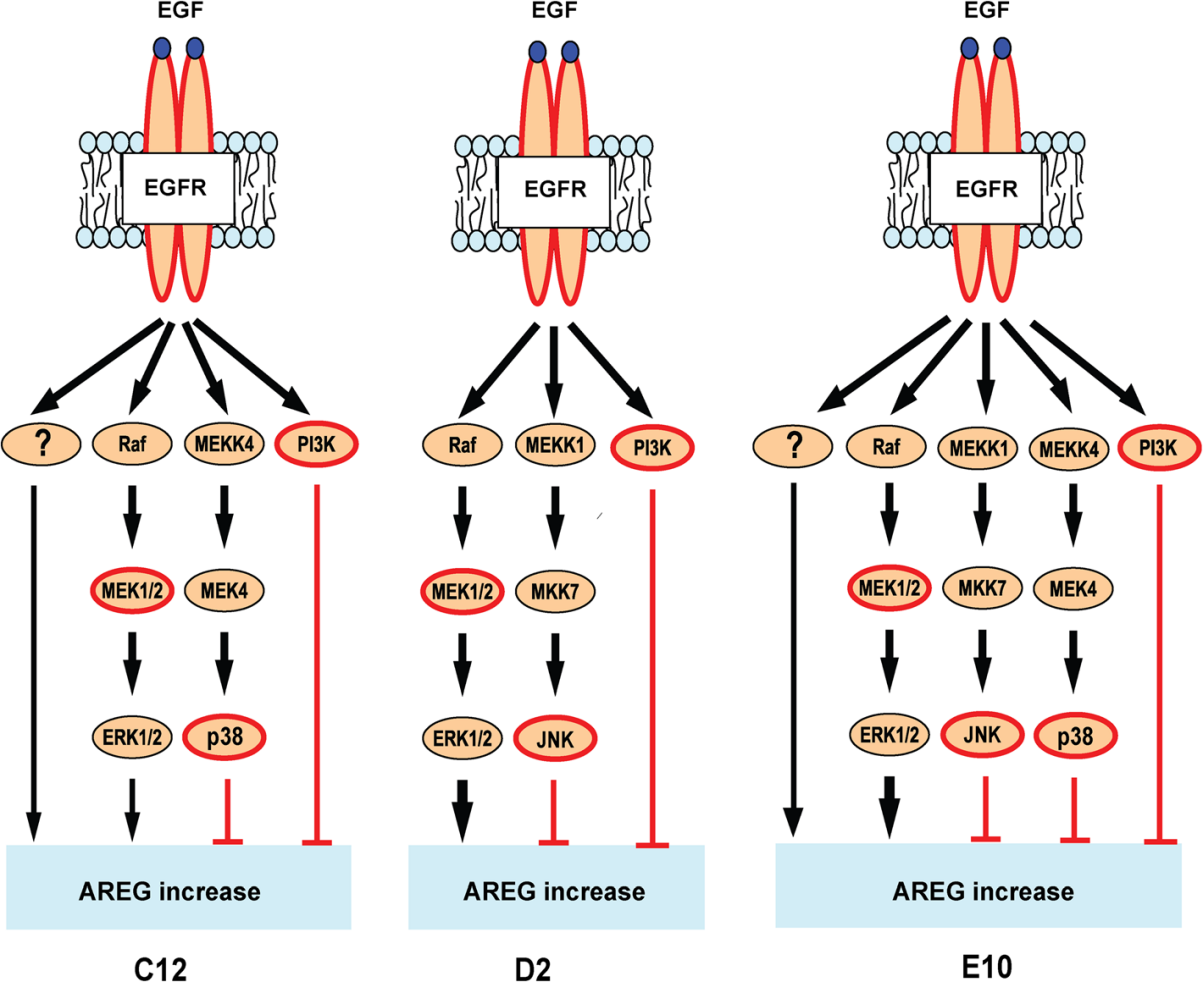
Signaling pathways in EGF-induced AREG mRNA expression. EGF-induced AREG expression was mediated by the extracellular signal-regulated kinase 1/2 (ERK1/2) in three cell lines, however only partially in the C12 and E10. AREG induction was seemingly mediated through an additional, unknown pathway (denoted “?”) in these cell lines. The two stress MAPKs negatively regulated the EGF-induced AREG expression, p38 in the C12 and E10 cell lines, and JNK in the D2 and E10 cell lines. Additionally, EGF-induced AREG expression was negatively regulated by PI3K in all three cell lines

Interestingly, although EGF stimulation increased AREG protein secretion in the cancer cell lines, in a human gingival progenitor cell line (HGEPp) and in a transformed immortalized keratinocytes cell line (HaCaT), it did not increase AREG secretion in normal primary oral keratinocyte cell lines (Gao et al., unpublished data). Thus, EGF-induced AREG secretion may be a very tightly regulated process in normal cells presumably through the same three intracellular signaling pathways (p38, JNK, and PI3K) that regulated EGF-induced AREG secretion in OSCC and transformed cells. The transformed and the OSCC cell lines had lost at least one of these regulatory pathways (Fig. 7), allowing EGF-stimulation to increase AREG production. Losing one of these AREG-regulatory pathways may, therefore, be a fundamental process in carcinogenesis.

Both EGF- and HBEGF- induced AREG may increase cell proliferation, anchorage-independent growth, and reduce apoptosis in an almost autocrine manner, as shown for hepatocellular carcinoma [33]. Furthermore, EGFR-stimulation induces COX-2 and PGE2 production in OSCC cell lines [34], which may further increase AREG expression due to EGFR-cross activation, as shown for colon cancer cell lines [35]. EGFR activation induced phosphorylation of the PI3K-, and the three MAPK-pathways with different roles in the EGF-induced AREG expression. MEK inhibition substantially reduced EGF-induced AREG mRNA and protein expression in all three cell lines, illustrating a more total ERK1/2 pathway dependency than previously observed in human skin organ cultures [36]. In contrast to insulin-induced AREG mRNA expression in RT4 bladder cancer cells [37], PI3K inhibition increased EGF-induced AREG mRNA expression, but not protein secretion. Thus, PI3K may regulate AREG mRNA transcription and/or intracellular AREG trafficking [35].

In addition to PI3K, the two stress-activated MAPKs, JNK and p38, negatively regulated EGF-induced AREG mRNA. This could have been through negative regulation of the ERK1/2 pathway, either through an unknown mechanism (JNK) or through activation of the phosphatase PP2A (p38) [38]. Interestingly, cisplatin treated breast cancer cells overexpressed phospho-ERK1 and expressed increasingly more AREG as cisplatin resistance developed [17]. In addition, surviving cisplatin sensitive ovarian cancer cells had sustained JNK and p38 activation after cisplatin treatment [39]. However, despite several reports showing a potential link between cisplatin resistance and increased AREG expression in various cancers [17, 18], AREG expression levels did not correlate to cisplatin resistance in the current 11 OSCC cell lines. Furthermore, exogenous AREG did not increase cisplatin resistance in the sensitive cell lines, and the two *in house* made cisplatin-resistant cell lines had either increased (C12cis) or decreased (D2cis) AREG mRNA expression. No other EGFR ligands were increased in the two *in house* made cisplatin resistant cell lines (unpublished data), suggesting that cisplatin resistance was not mediated by any of these growth factors alone. Although most of the cis-/carbo-platin treated patients had advanced disease (TNM stage IV), patients with high AREG expressing tumors had, in particular, perineural, lymphovascular and nodal extracapsular infiltration. This may reflect AREG induced increased cancer cell motility, migration and infiltrative growth, which may explain the poor prognosis as AREG did not inhibit cisplatin cytotoxicity.

## Conclusions

Increased tumorous mRNA expression of up to four EGFR ligands was progressively associated with poor prognosis in HNSCC. Thus, this may be a new prognostic biomarker in monitoring patients as it was superior to the TNM staging system. EGF stimulated AREG production was tightly regulated in normal and transformed cell lines, suggesting that failure to control EGFR induced AREG expression may be a crucial step in carcinogenesis and cancer progression. Inhibiting EGF-induced AREG expression may be a novel strategy in HNSCC treatment.

## Abbreviations

AREG: Amphiregulin
BTC: Beta-cellulin
EGF: Epidermal growth factor
ELISA: Enzyme linked immunoassay
EPGN: Epigen
EREG: Epiregulin
ERK: Extracellular signal-regulated kinase
HBEGF: Heparin-binding EGF-like growth factor
HNSCC: Head and neck squamous cell carcinoma
JNK: c-Jun N-terminal kinase
MAPK: Mitogen activated protein kinase
MEK: MAPK/ERK kinase
mRNA: Messenger RNA
OSCC: Oral squamous cell carcinoma
PI3K/Akt: Phosphoinositide 3-kinase/protein kinase B
RT-PCR: Reverse transcriptase polymerase chain reaction
TBP: TATA-box binding protein
TGF-α: Transforming growth factor-α
